# Genomic Epidemiology of West Nile Virus in Paris

**DOI:** 10.1001/jamanetworkopen.2025.59588

**Published:** 2026-02-16

**Authors:** Raphaelle Klitting, Mathilde Gondard, Laura Pezzi, Camille Victoire Migné, Rachel Bellone, Grégory L’Ambert, Teheipuaura Helle, Antoine Mignotte, Raquel Gutiérrez-Climente, Guillaume Lacour, Julien Mocq, Nazli Ayhan, Federico Lucchese, Géraldine Piorkowski, Marine Dumarest, Rayane Amaral, Karine Bollore, Jeanne Hanin, Olivier Courot, Edouard Hirchaud, Véronique Beven, Yannick Blanchard, Anais Karch, Georges Jakerian, Guillaume André Durand, Gilda Grard, Nicolas Herbreteau, Alexandre Duvignaud, Serafin Gutierrez, Lydéric Aubert, Arnaud Cannet, Marion Parisey, Yannick Simonin, Denis Malvy, Marie-Claire Paty, Nelly Fournet, Florian Franke, Syria Laperche, Pierre Gallian, Anna-Bella Failloux, Xavier de Lamballerie, Albin Fontaine, Gaëlle Gonzalez

**Affiliations:** 1 Centre National de Référence des Arbovirus, National Institute of Health and Medical Research-Institut de Recherche Biomédicale des Armées, Marseille, France; 2 Unité des Virus Émergents, Aix-Marseille Univ, Università di Corsica, IRD 190, National Institute of Health and Medical Research 1207, Institut de Recherche Biomédicale des Armées, Marseille, France; 3 Agence nationale de sécurité sanitaire de l’alimentation, de l’environnement et du travail, Institut national de recherche pour l’agriculture, l’alimentation et l’environnement, Ecole Nationale Vétérinaire d’Alfort, UMR Virologie, Laboratoire de Santé Animale, Maisons-Alfort, France; 4 Department of Virology, Arboviruses and Insect Vectors, Institut Pasteur, Paris, France; 5 Entente interdépartementale pour la démoustication du littoral méditerranéen , Montpellier, France; 6 Altopictus, Pérols, France; 7 Pathogenesis and Control of Chronic and Emerging Infections, Université de Montpellier, National Institute of Health and Medical Research, Montpellier, France; 8 Ingénierie et Analyse en Génétique Environnementale, Montpellier, France; 9 Agence nationale de sécurité sanitaire de l’alimentation, de l’environnement et du travail, Laboratory of Ploufragan, Unit of Viral Genetics and Biosafety, Ploufragan, France; 10 Agence Régionale de Démoustication, Rosny-sous-Bois, France; 11 Agence Régionale de Santé, Île-de-France, France; 12 Department of Infectious Diseases and Tropical Medicine, CHU Bordeaux, France; National Institute for Health and Medical Research, Bordeaux, France; 13 UMR 1219, Research Institute for Sustainable Development (IRD) EMR 271, Bordeaux Population Health Research Centre, University of Bordeaux, Bordeaux, France; 14 Animal, Santé, Territoires, Risques et Écosystèmes, Centre de coopération internationale en recherche agronomique pour le développement, Institut national de recherche pour l’agriculture, l’alimentation et l’environnement, University of Montpellier, Montpellier, France; 15 Laboratory of Virology, Montpellier University Hospital, Montpellier, France; 16 Direction générale de la Santé, Centre de Crises Sanitaires, Paris, France; 17 Centre Hospitalier de Saint-Denis Hôpital Delafontaine, Pulmonology & Infectious Diseases, Saint Denis, France; 18 Santé publique France (French National Public Health Agency), Saint Maurice, France; 19 Etablissement Français du Sang, La Plaine Saint Denis, France; 20 Institut de Recherche Biomédicale des Armées-, Unité de virologie, Marseille, France

## Abstract

**Importance:**

West Nile virus (WNV), a mosquito-borne orthoflavivirus, represents an increasing public health threat in Europe. In July 2025, WNV lineage 2 (WNV-L2) was associated with the first autochthonous cases ever detected in the metropolitan area around Paris, France; understanding the dispersal dynamics and geographic origin of these emergence events is critical for public health preparedness.

**Objectives:**

To characterize the spatial and temporal dynamics of WNV circulation in France from 2022 to 2025 and to determine the origin of the virus lineages responsible for the 2025 emergence in the Paris metropolitan area.

**Design, Setting, and Participants:**

This study was a genomic epidemiology assessment combining human, veterinary, and entomological monitoring, with viral genome sequencing and time-resolved, bayesian phylogenetic analysis. Surveillance was conducted across metropolitan France, with a focus on areas with documented WNV circulation (Eastern and Western Mediterranean, South Atlantic, and Paris). Surveillance included patients with confirmed WNV infection, avian and equine cases, and mosquito collections.

**Exposures:**

Natural exposure to WNV through mosquito vectors in affected areas.

**Main Outcomes and Measures:**

Phylogenetic relationships between emergence events across regions in France were inferred using a bayesian approach.

**Results:**

Genomic data from this epidemiological assessment encompassed 52 WNV-positive samples (6 human, 21 veterinary, and 25 entomological samples). WNV-L2 was the only lineage detected in France between 2022 and 2025. In 2024, Western Mediterranean strains were closely related to those from 2023 from the French South Atlantic area, suggesting west-to-south introduction, whereas Eastern Mediterranean strains represented genetically distinct clades associated with Northern Italy. The phylogenetic analysis of 2025 WNV sequences from the Paris area revealed that the outbreak virus strains all grouped together and originated from the L2 clade of sequences from the South Atlantic area in the 2023 to 2024 period.

**Conclusions and Relevance:**

In this study, genomic data from an epidemiologic assessment of 52 WNV-positive samples highlight the combined influence of local virus maintenance and long-distance dispersal in shaping WNV circulation in France. Strengthened genomic surveillance across ecological and administrative boundaries would be essential to anticipate further viral spread, inform response strategies, and protect populations at risk.

## Key Points

**Question:**

What are the spatial and temporal dynamics of West Nile virus (WNV) circulation in France, and what is the virus lineage at the origin of the 2025 emergence in the Paris area?

**Findings:**

In this study, conducted between 2022 and 2025 and integrating human, veterinary, and entomological surveillance across France, only lineage 2 was detected among the 52 WNV-positive samples analyzed. The phylogenetic analysis linked Mediterranean virus strains to those circulating in the South Atlantic over previous years and found that the 2025 Paris outbreak was caused by virus strains also originating from the South Atlantic sublineage.

**Meaning:**

These findings suggest WNV expansion in France is sustained by both local maintenance and long-distance dispersal across regions, underscoring the need for strengthened genomic surveillance to anticipate future emergence.

## Teaser

This study of human, veterinary, and entomological surveillance investigates the spatial and temporal dynamics of West Nile virus circulation in France and its 2025 origin in the Paris metropolitan area.

## Introduction

West Nile virus (WNV) is an orthoflavivirus primarily transmitted by *Culex* species mosquitoes and maintained in an enzootic cycle involving birds as amplifying hosts. Spillover infections can occur in mammals, including horses and humans, which are considered dead-end hosts and may develop neuroinvasive disease. Since its initial isolation in Uganda in 1937, WNV has spread globally, becoming the most widespread arbovirus in the world and causing recurrent outbreaks in Africa, the Middle East, Europe, South America, and North America.

**Figure 1.**
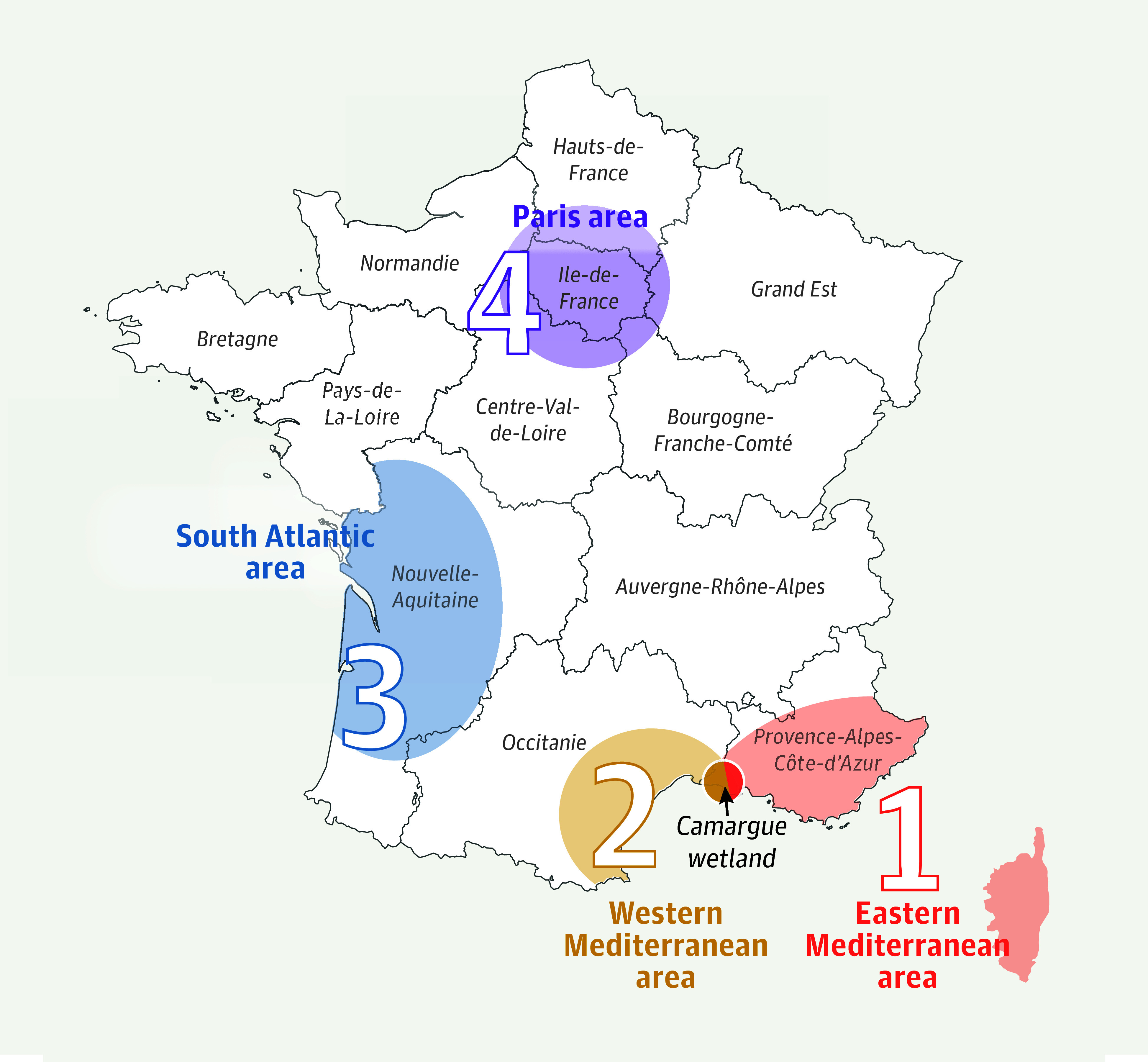
Geographic Distribution of West Nile Virus Circulation Areas in France West Nile virus has historically been reported in the eastern Mediterranean area (1; most of Provence-Alpes-Côte d’Azur and Corsica) and the western Mediterranean area (2; the coast of Occitanie), connected by the Camargue wetland, which overlaps with both regions. Most recent detections have occurred in the south Atlantic area (3; coastal Nouvelle-Aquitaine) and, most recently, in the Paris region (4; Île-de-France).

**Figure 2.**
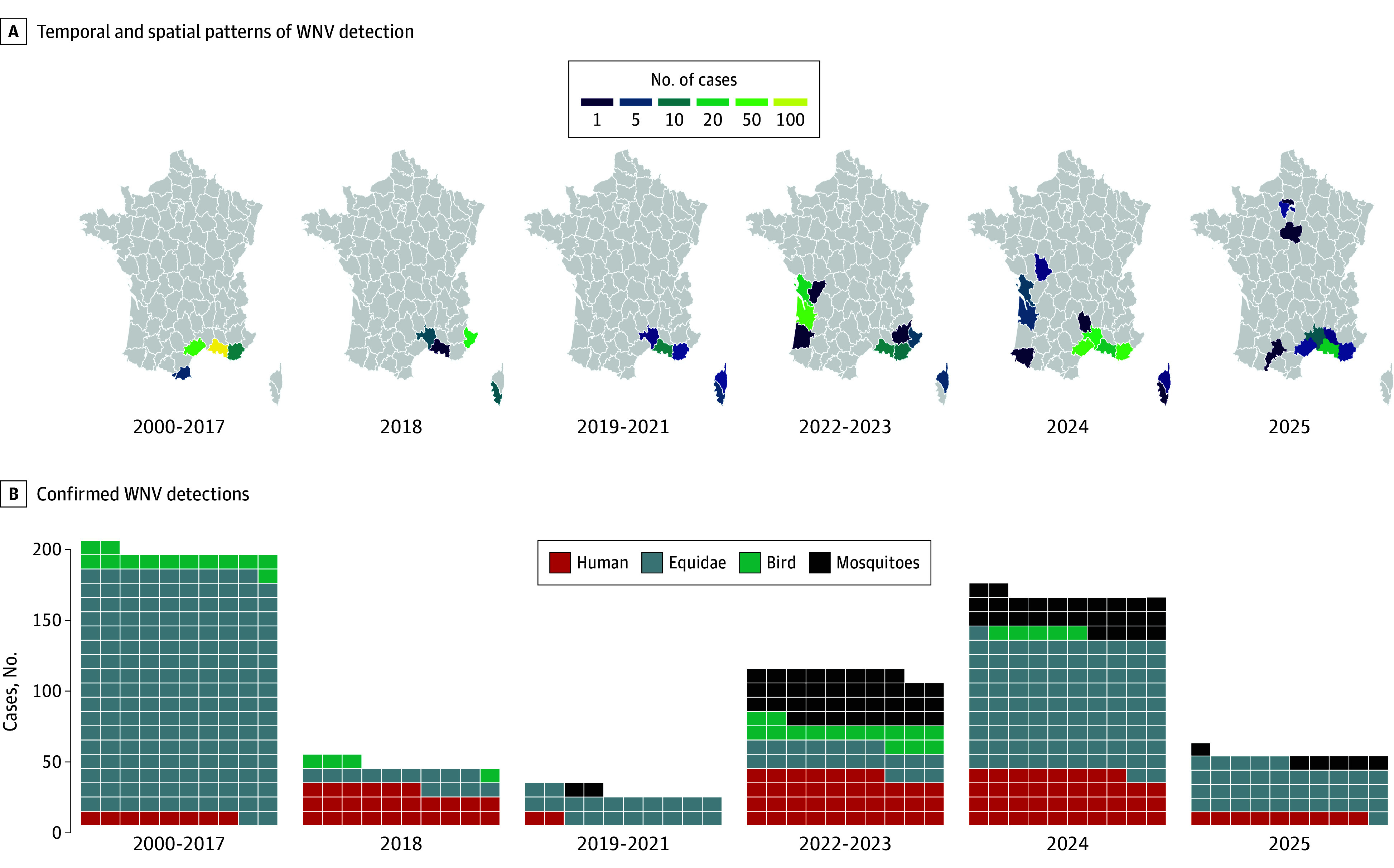
Temporal and Spatial Patterns of West Nile Virus (WNV) Detections in France Across Different Host and Vector Systems A, Choropleth map shows French departments, shaded according to the cumulative number of WNV detections across all 3 surveillance sources. B, The tile plot shows the number of confirmed detections in humans, equidae, and through molecular xenomonitoring (positive mosquito excreta) for each period.

In metropolitan France, WNV circulation was historically restricted to the Camargue wetland and adjacent Mediterranean areas (Figures 1 and 2). The virus was first detected there in 1962 as WNV lineage 1 (WNV-L1).^1^ WNV lineage 2 (WNV-L2) was first reported more than 5 decades later near the Italian border in the Eastern Mediterranean area in 2018, and has been repeatedly reported there since.^2^ This lineage subsequently expanded westward and northward, with WNV-L2 strains identified in both the Eastern Mediterranean area (2018-2023) and the South Atlantic area (2022-2023).^3^

Between 2023 and 2024, integrated One Health surveillance—combining data from medical, veterinary, and entomological surveillance in keeping with the One Health holistic approach to human and animal health— revealed concurrent WNV activity in Eastern and Western Mediterranean areas as well as in the South Atlantic region, involving human, equine, avian, and mosquito detections.^3,4,5^ In 2025, WNV activity persisted in Southern France^6^ and, for the first time, WNV-L2 was detected in humans, mosquitoes, and nonhuman vertebrates in the Paris area^7^—far beyond previously affected regions (eFigure in Supplement 1). The detection of human neuroinvasive cases, positive mosquito excreta, and equine infections^8^ across several weeks confirmed active local transmission in and around metropolitan Paris.

These recent shifts in WNV spatial distribution raise critical questions about viral dynamics, particularly the routes of circulation between impacted areas. Despite active surveillance, the lack of comprehensive genomic data has limited our ability to reconstruct viral spread within France and its connections to broader European transmission networks. Here, we combine genomic sequencing with nationwide surveillance data from 2022 to 2025 to decipher the spatiotemporal dynamics of WNV in France, delineate regional circulation patterns, and trace the origins of the lineage recently emerging in the Paris area.

## Methods

Human samples included in this study were collected as part of the national public health surveillance program coordinated by the National Reference Centre (NRC) for Arboviruses and supervised by Santé publique France. As these samples constituted epidemiological surveillance records, consultation with an ethics committee was not required. Samples were selected from human plasma and serum specimens routinely received by the Arbovirus NRCs for standard diagnostic and expert activities. All samples transferred to the NRC were submitted with an accompanying form including the patient’s consent through a nonopposition clause. No additional clinical specimens were collected specifically for this study. Human samples and derived viral cultures were fully anonymized, ensuring no or minimal risk to patients, in accordance with the European General Data Protection Regulation and the requirements of the French National Commission on Informatics and Liberties.

### Sampling Design

Human samples used in this study were obtained either from symptomatic patients tested at the French NRC for Arboviruses (Marseille, France) or from asymptomatic blood donors detected through the French blood establishment (in departments where WNV transmission in humans has been documented, in accordance with recommendations from the Haut Conseil de la Santé Publique). Animal samples (including dead birds and symptomatic horses) were collected as part of WNV surveillance conducted by the French National Reference Laboratory for WNV (Agence nationale de sécurité sanitaire de l’alimentation, de l’environnement et du travail, Maisons-Alfort, France). Mosquitoes and their excreta were collected in the context of entomological surveillance performed near confirmed human or animal cases, or in areas considered at risk for WNV circulation. Additionally, in 2025, mosquito excreta were collected within the framework of the Émergence de Maladies vectorielles liées au moustique tigre project (known as EMa-Tigre) funded by the Fondation Crédit Mutuel Alliance Fédérale and coordinated by the Arbovirus et Insectes Vecteurs team at the Institut Pasteur, Paris. The project aims to identify arboviruses circulating in mosquito populations in France.

### Mosquito and Mosquito Excreta Collection: Molecular Xenomonitoring Strategy

Molecular xenomonitoring (MX) was conducted using modified BG-Sentinel or BG-Pro traps (BGS, Biogents AG), for a minimum of 24 hours and up to 7 consecutive days. The protocol was adapted from Bigeard et al.^3^ Briefly, in these modified traps, the standard collecting bags were replaced with a 3-dimensional–printed MX adapter positioned beneath the intake funnel. Aluminum foil was placed at the bottom of the adapter to collect mosquito excreta during the trapping period. Mosquito excreta were subsequently tested by reverse transcriptase–quantitative polymerase chain reaction (RT-qPCR). When excreta samples yielded a positive result, the corresponding mosquitoes were analyzed individually. A detailed description of the full MX protocol is provided in eMethods in Supplement 1.

### Molecular Screening of WNV Genome

Samples from symptomatic human cases (plasma, serum, whole blood, and urine) were tested by RT-qPCR using WNV primers and probes (reference 001K-05424) and a synthetic positive control (reference 001K-05425) provided by the European Virus Archive Marseille on Panther Fusion (Hologic). Plasma from asymptomatic blood donors was screened using the Cobas WNV assay on the Cobas 8800 (Roche).

Mosquito excreta (rinsed from aluminum foil) and homogenized mosquitoes were extracted and tested by RT-qPCR (CFX; Bio-Rad) with the same primers and/or probes used for symptomatic human cases. Selected excreta from Western Mediterranean areas were analyzed by digital PCR.

Animal samples (horse brain and bird organs or swabs) were processed in Dulbecco modified Eagle medium, and RNA was extracted and screened by duplex RT-qPCR (AgPath-ID kit on QS5 [Thermo Fisher]) targeting WNV and β-actin, with primers and probes from the European Union Reference Laboratory for Equine Diseases. The complete protocol for molecular screening of excreta, mosquitoes and animal samples is provided in eMethods in Supplement 1.

### Virus Sequencing

For animal samples sequencing at the National Reference Laboratory, 2 distinct approaches were employed. For a subset of samples, RNA was directly used for RNA-seq library preparation using the Illumina Stranded Total RNA Prep kit, with indexing performed using IDT for Illumina indexes. Libraries were sequenced on a NextSeq 2000 system (Illumina). Other samples were processed using an amplicon-based sequencing approach as previously described,^9^ with primers specifically designed to generate 400–base pair (bp) overlapping amplicons covering the full genome of WNV-L2 and provided by the European Union Reference Laboratory for Equine Diseases and sequenced on either Illumina or Oxford Nanopore Technologies platforms.

WNV genomes from human and mosquito (excreta or whole mosquito) samples were sequenced at the NRC using an amplicon-based sequencing approach with a set of 8 overlapping amplicons, previously used for WNV sequencing.^3^ Sequencing was conducted on the Ion S5 System (Thermo Fisher Scientific) according to the manufacturer’s instructions.

Detailed protocols for sequencing of human, mosquito and animal samples are available in eMethods in Supplement 1. All genomic sequences were made publicly available on GenBank either directly or via the Pathoplexus platform.^10^

### Phylogenetic Analysis

Publicly available sequences representative of the genetic diversity of WNV-L2 were retrieved from the pathoplexus database (SeqSet).^11^ Open reading frame coding regions were aligned using MAFFT version 7.511 (Research Institute for Microbial Diseases), and phylogenetic relationships between public WNV genomes and the sequences generated in this study were inferred with IQ-Tree version 1.6.12 (IQ-Tree Development Team) using the best-fit nucleotide substitution model and ultrafast bootstrap approximation (UFBoot2 1000 replicates); the bootstrap support threshold was defined as 95. On the basis of the initial phylogenetic tree, we selected a subset of 218 sequences representative of WNV-L2 combined with WNV-L2 sequences from France (2018-2025). We reconstructed a time-scaled phylogeny with BEAST version 1.10.5,^12^ after selecting the best-fit model (eTable 1 in Supplement 1), we ran 2 Markov chain Monte Carlo chains of 500 million states and used Tracer version 1.753^13^ for inspecting convergence and mixing, ensuring that estimated sampling size values were all greater than 200. All alignment, xml, and tree files for this study are available on a public github repository.^14^

### Statistical Analysis

The analyses were descriptive and based on genomic sequencing and phylogenetic inference; therefore, no statistical tests or significance levels were applied besides those relative to the phylogenetic analysis, which were previously detailed in the Methods section. Data analyses were performed using R version 4.4.2 (R Project for Statistical Computing) and Python 3.13.2 (Python Software Foundation), implemented through Snakemake pipelines and the RStudio integrated development environment.

## Results

**Figure 3.**
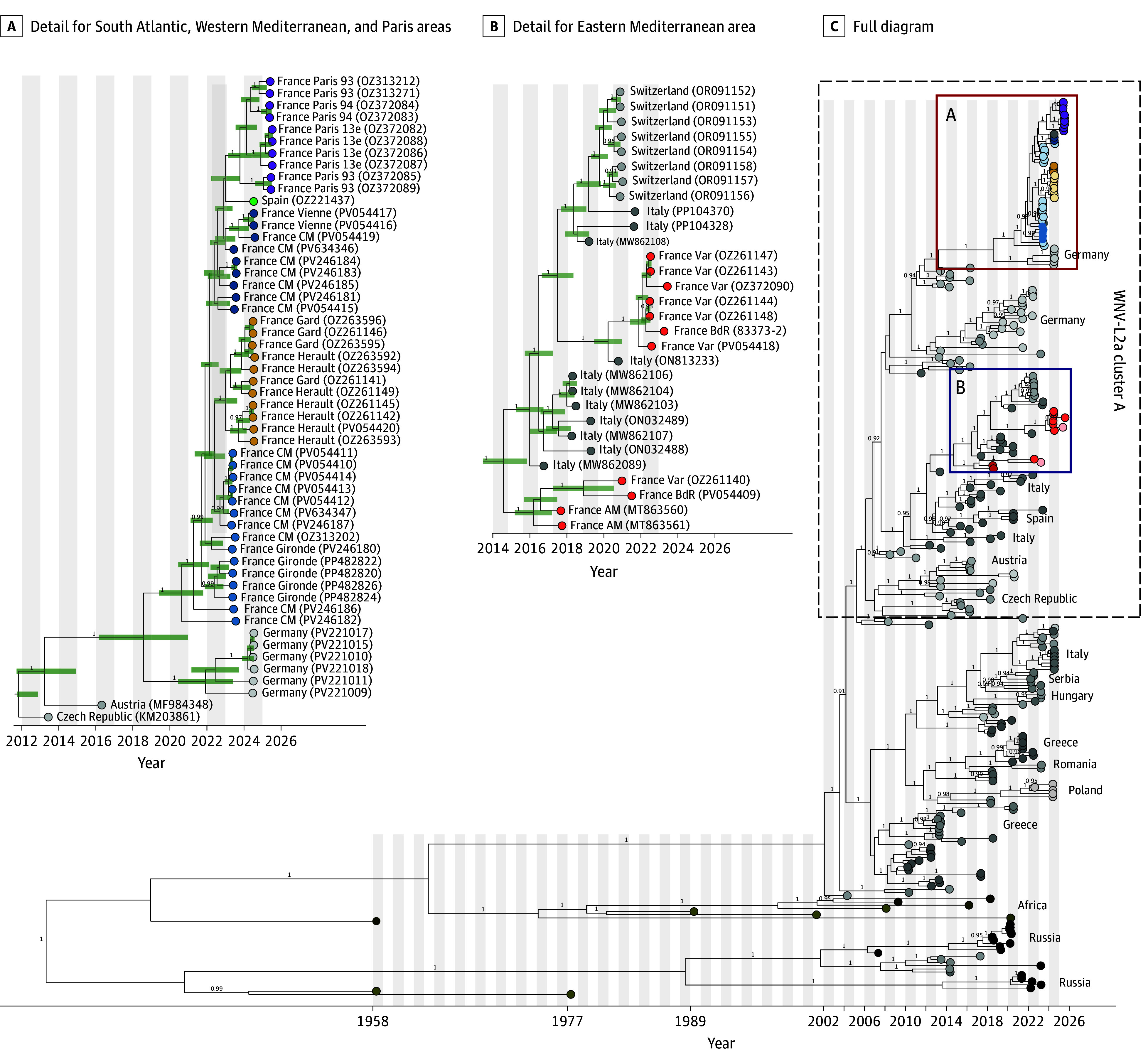
Phylogenetic Relationships of West Nile Virus (WNV) Lineage 2 Circulating in France Maximum clade credibility time-scaled phylogenies using 266 WNV-L2 sequences from Europe, including 52 generated in this study. Posterior supports greater than 90% are shown. The main tree highlights WNV-L2a subcluster A, as defined by Lu et al.^15^ Two subclades within this cluster are displayed in detail: subclade A (sequences from the south Atlantic, western Mediterranean, and Paris regions) and subclade B (sequences from the eastern Mediterranean region). In the zoomed panels, tree tips are color-coded by geographic origin, with colors matching known areas of WNV circulation in France.

A total of 52 samples (6 human, 2 equine, 19 avian, and 25 entomological) were available for analysis. The final dataset included 8 sequences from the Eastern Mediterranean area, 11 from the Western Mediterranean area, 23 from the South Atlantic area, and 10 from Paris area (eTable 2 in Supplement 1). All sequences belonged to WNV-L2, indicating that this lineage has now spread across a large part of Metropolitan France (Figure 3).

To investigate potential relationships between WNV strains from the 4 areas where the virus was detected in recent years, we inferred a time-resolved phylogeny using bayesian inference, based on a dataset combining our sequences and 218 sequences representative of the diversity within WNV-L2 public genomes obtained from the PathoPlexus database.^16^ Previous phylogenetic analysis of WNV genomes from France showed that WNV strains from the Eastern Mediterranean and South Atlantic areas formed distinct clades within the subcluster A of WNV-L2^15^ (also called Central or South-West European^3^). According to our phylogenetic inference, we found that WNV sequences from the Eastern Mediterranean area were distinct from earlier sequences detected in 2018, 2022, and 2023. They still clustered separately from those from the South Atlantic, grouping with recent sequences from Northern Italy (Piemonte, 2022-2023), and Switzerland (2022). On the basis of our phylogenetic dating, we estimated that the 2024 Eastern Mediterranean clade emerged around October 2023 (95% highest posterior density [HPD], April 11, 2023, to May 2, 2024), whereas the clade encompassing sequences from 2018 to 2023 dated back to November 2016 (95% HPD, August 21, 2015, to January 1, 2018).

When examining the sequences from the Western Mediterranean area in 2024, we found that they formed a unique cluster nested within a larger clade encompassing all sequences from the South Atlantic area in the period from 2022 to 23 as well as a single sequence recently identified in Spain (2024). We estimated that the Western Mediterranean area clade emerged around March 2023 (95% HPD, July 1, 2022, to October 4, 2023), whereas the larger and more genetically diverse South Atlantic clade dated back to approximately October 2020 (95% HPD, June 27, 2019, to November 8, 2021).

When analyzing the sequences obtained from human cases and mosquitoes in the Paris area in 2025, they formed a single cluster rooted within the larger South Atlantic clade, separately from sequences from the Eastern Mediterranean area. Our phylogenetic dating estimates suggest that the Paris clade likely emerged before the end of 2024 (95% HPD, May 5, 2023, to August 23, 2024).

## Discussion

In 2024, WNV circulation intensified across Europe, with 19 countries reporting a total of 1436 locally acquired human cases.^17^ Albania experienced its largest recorded outbreak, while Poland reported its first locally acquired human infection.^17^ In France, WNV-L2 was detected in 3 distinct regions, including the Camargue wetland—a territory historically associated with WNV-L1 activity.^18^ At present, WNV-L2 is becoming predominant in France, although WNV-L1 continues to circulate in Italy, where both lineages coexist.^19^ WNV, which was once confined to circulation in rural wetland areas, is now spreading into densely populated urban centers, including some of the largest cities in France.^3,7^ Several studies now clearly document the progressive westward and northward expansion of WNV-L2 across Europe. In Italy, national surveillance data from 2011 to 2021 demonstrate that WNV-L2 has gradually supplanted L1, with sharp increases in case numbers and geographic spread during the 2015 to 2018 period and again in 2022.^20^ The factors underlying this trend are likely multiple, including increased replicative and transmission fitness. A recent continent-wide phylodynamic analysis further showed that a distinct subcluster of WNV-L2, previously referred to as the Central-Eastern European clade^21^ and subsequently renamed WNV-2a, accounts for approximately 73% of all available European WNV-L2 genomes.^15^ Phylodynamic dating places the emergence of WNV-2a around July 2006 (95% HPD, January 2005 to March 2007) in Austria. Notably, all WNV-L2 sequences recently detected in France—including those from the south Atlantic area in the 2022 to 2023 period, the Western and Eastern Mediterranean regions in 2024, and Paris in 2025—belong to the WNV-2a subcluster A. In this study, we further characterize 2 sublineages: WNV-L2a A-1, which includes viruses circulating in the South Atlantic, Western Mediterranean, and Paris areas, and WNV-L2a A-2 (Figure 3), which appears to be currently restricted to the Eastern Mediterranean area.

Because WNV circulation in Europe relies on multiple distinct mechanisms that cannot be determined on the basis of virus occurrence data alone—recurrent introductions, long-distance dispersal, and potential local overwintering—genomic data are essential to link circulating virus strains across regions. Specifically in France, our results highlight clear differences in WNV dynamics between the Western and South Atlantic as well as the Eastern Mediterranean regions. On the one hand, the 2024 WNV-L2 sublineage circulating in the Eastern Mediterranean is genetically distinct and appears to result from recent importations from Italy, where WNV-L2 has circulated intensely since the early 2000s.^22^ Previous sequences collected in southeastern France between 2018 and 2023 also cluster with Italian strains, suggesting repeated introductions from Northern Italy into the Eastern Mediterranean area.

On the other hand, our analyses show that local WNV maintenance over several years on the South Atlantic coast likely fueled WNV circulation in other areas of France. Strains from the South Atlantic form a unique diverse clade with an estimated most recent common ancestor dating back to the end of 2020, indicating cryptic circulation locally—or in an intermediate location—for over a year before the first detection of WNV in this area. Our results show that the Western Mediterranean WNV-L2 strains detected in 2024 are closely related to those from the South Atlantic area in 2023 to 2024. Although limited by the sparse WNV genomic sampling in Southern France and neighboring Spain, our inference suggests that WNV was introduced from the South Atlantic area (or an unsampled area that would also fuel virus circulation in the South Atlantic) into the Western Mediterranean area a few years ago (prior to October 2023). Furthermore, our phylogenetic analysis also links the WNV-L2 strains detected in the Paris area in 2025 to the South Atlantic clade, suggesting that this area may act as a hub for the dispersal of WNV to both the South and the North of the country. Moreover, the genetic similarity between South Atlantic strains and a virus detected in southern Spain in 2024 indicates likely virus exchanges between southwestern France and that neighboring country.^23^

The mechanisms underlying WNV persistence, spread, and emergence in Europe remain poorly understood. Previous studies have highlighted the role of climatic conditions, land use, and bird migration in shaping WNV dynamics.^15,24,25,26,27^ Elevated temperatures are particularly influential, as they can enhance viral genetic diversity by increasing mosquito survival, shortening the extrinsic incubation period, and extending the transmission season, creating conditions more favorable to the onset of outbreaks. In addition, the level of herd immunity in local bird reservoirs appears to modulate viral amplification and circulation.^24^ We observed substantial WNV genetic diversity in the South Atlantic area—comparatively to other recent hotspots of WNV circulation in France—suggesting that this region could act as a source fueling viral emergence in other areas in subsequent years. Notably, in 2022, this area experienced a historic wildfire—one of the largest in the past 50 years—with approximately 25 000 hectares burned.^28^ Such an extreme ecological disturbance may have disrupted natural buffer zones and altered local WNV transmission dynamics by reshaping bird and mosquito habitats, potentially facilitating interactions between enzootic cycles and urban environment.

Molecular epidemiology, by tracking the genetic evolution of WNV, provides a powerful means to monitor the geographic distribution of viral variants, their temporal dynamics, their origins, and to delineate areas of sustained transmission. WNV often establishes persistent endemic circulation in the regions where it emerges—well illustrated by the long-term establishment of WNV-L1 in North and South America. These genomic insights support intensified early-season surveillance (April to June) in entomological and avian compartments within previously affected areas. Routine sequencing of even a subset of positive samples would enable near real-time clade tracking, confirm overwintering events, and improve anticipation of transmission risk. Here we assess virus spread by sampling over multiple components of the transmission chain—human, equine, avian, and mosquito hosts—highlighting the importance of cross-sectoral data integration, shared alert thresholds, and coordinated outbreak investigations to better study and respond to emerging zoonoses. Together, this genomic framework strengthens national preparedness by guiding targeted surveillance, early detection, and One Health response strategies in a context of expanding WNV activity across Europe.

### Limitations

This study has limitations that should be mentioned. The unforeseen emergence of WNV in Paris highlights major gaps in our understanding of the ecological and epidemiological mechanisms underlying viral persistence and spread, including the role of resident and migratory birds, vector movement, and climatic variability. These gaps are reflected in our phylogenetic dating, which suggests that WNV may have circulated unnoticed in the South Atlantic area for multiple years before causing human cases. Addressing these uncertainties is essential to anticipate future geographic expansions and to adapt surveillance and public health strategies accordingly. Notably, surveillance of wild bird populations for WNV remains a major weakness, representing a critical area for improvement in future monitoring efforts. Furthermore, the sparse genomic sampling of WNV in France and neighboring countries limits our ability to decipher with certainty the spatial spread of the virus within France and with neighboring countries.

## Conclusions

This study confirmed the widespread circulation of WNV-L2 in France. These findings underscore the urgent need to expand and coordinate genomic surveillance across Europe, especially in both historically endemic regions and newly affected areas. Additionally, enhancing genomic monitoring in Africa—especially along key migratory bird flyways—is essential to understand the origins of WNV lineages introduced into Europe, assess the risk of reintroduction, and anticipate future emergence events.

## Notes

Supplement 1. eFigure. Map of Paris area showing the temporal dynamics of WNV detections in humans, horses, and mosquitoes eTable 1. Model comparison and marginal likelihood estimation for West Nile virus lineage 2 (WNV-L2) phylogenetic analyses eTable 2. Metadata for WNV-L2 sequences obtained from positive samples collected in France between 2022 and 2025 eMethods. eReferences

Supplement 2. Data Sharing Statement
