# Errors in Results and Discussion

**DOI:** 10.1001/jamanetworkopen.2026.5966

**Published:** 2026-03-11

In the Original Investigation titled “Genomic Epidemiology of West Nile Virus in Paris,”^1^ published February 16, 2026, time to the most recent common ancestor was incorrectly estimated by including the stem of the corresponding clade. These values have now been corrected to estimates not including the stem. This correction does not affect the overall structure of the phylogeny, inferred relationships between clades, nor the main conclusions of the study, but it does modify the estimated dates reported in the Results and corresponding interpretations in the Discussion. This article has been corrected.^1^
