# Supplementary material for: Genomic Epidemiology of West Nile Virus in Paris
Source: JAMA Netw Open. 2026 Feb 16;9(2):e2559588. doi: 10.1001/jamanetworkopen.2025.59588 (PMC12910402; doi:10.1001/jamanetworkopen.2025.59588)
Supplement: Supplement 1. — eFigure. Map of Paris area showing the temporal dynamics of WNV detections in humans, horses, and mosquitoes eTable 1. Model comparison and marginal likelihood estimation for West Nile virus lineage 2 (WNV-L2) phylogenetic analyses eTable 2. Metadata for WNV-L2 sequences obtained from positive samples collected in France between 2022 and 2025 eMethods. eReferences [file jamanetwopen-e2559588-s001.pdf]

Supplemental Online Content

Klitting R, Gondard M, Pezzi L, et al. Genomic epidemiology and the emergence of West Nile Virus in France. *JAMA Netw Open.* 2026;9(2):e2559588. doi:10.1001/jamanetworkopen.2025.59588

eFigure: Map of Paris area showing the temporal dynamics of WNV detections in humans, horses, and mosquitoes

eTable 1 : Model comparison and marginal likelihood estimation for West Nile virus lineage 2 (WNV-L2) phylogenetic analyses

eTable 2 : Metadata for WNV-L2 sequences obtained from positive samples collected in France between 2022 and 2025

eMethods

eReferences

This supplemental material has been provided by the authors to give readers additional information about their work.

© 2026 Klitting R et al. *JAMA Network Open*.

eFigure 1: Map of Paris area showing the temporal dynamics of WNV detections in humans, horses, and mosquitoes.

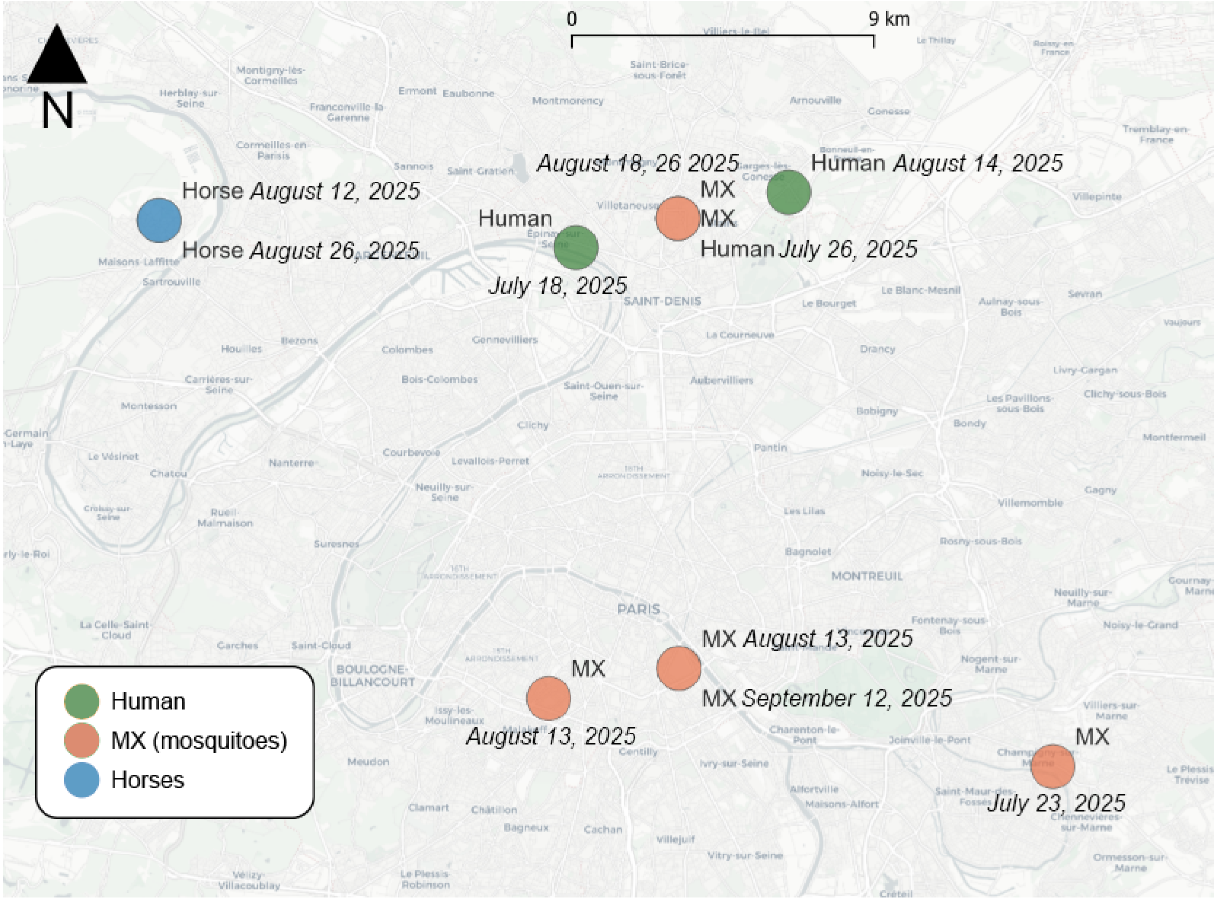

© 2026 Klitting R et al. *JAMA Network Open*.

eTable 1: Model comparison and marginal likelihood estimation for West Nile virus lineage 2 (WNV-L2) phylogenetic analyses. Analyses were performed on a subset of 218 WNV-L2 sequences from Cluster A. Two substitution models (HKY+G4 and SRD06) were combined with three coalescent priors (constant, exponential growth, and Bayesian skygrid) under an uncorrelated lognormal relaxed clock. Single MCMC chains of 100 million states were run, with marginal likelihoods estimated using path sampling (PS) and stepping-stone sampling (SS). For each model, the table reports estimated ages (median and 95% HPD) of key ancestral nodes (NAO, Occitanie, Var 2024, PACA). The SRD06 substitution model combined with a Bayesian skygrid prior showed the best fit, based on log marginal likelihood comparisons.

Substitution model	Tree prior	age NAO (median)	95%HPD	age (Occitanie)	95%HPD	age Var 2024	95%HPD	age PACA	95%HPD	Log marginal likelihood (PS)	Log marginal likelihood (SS)
**HKY+G4**	Constant	2013.4157	[2011.7743; 2015.3294]	2022.1177	[2021.2156; 2022.8565]	2021.7731	[2020.6054; 2022.7077]	2015.06	[2013.567; 2016.3796]	-47300.54	-47316.09
**SRD06**	Constant	2013.4651	[2011.9001; 2015.3571]	2022.13	[2021.2343; 2022.9017]	2021.7729	[2020.6402; 2022.724]	2015.13	[2013.5625; 2016.424]	-46087.37	-46100.61
**HKY+G4**	Exponential growth	2013.4185	[2011.8125; 2015.2932]	2022.1146	[2021.2484; 2022.8709]	2021.7774	[2020.621; 2022.7265]	2015.10	[2013.6114; 2016.4146]	-47306.97	-47318.40
**SRD06**	Exponential growth	2013.4228	[2011.8039; 2015.2327]	2022.1056	[2021.2229; 2022.9118]	2021.78	[2020.6012; 2022.7098]	2015.06	[2013.6242; 2016.4652]	-46088.45	-46096.82
**HKY+G4**	Skygrid	2013.1316	[2011.4887; 2015.137]	2022.6128	[2021.8981; 2023.2126]	2021.8806	[2020.7493; 2022.7892]	2014.90	[2013.356; 2016.4137]	-47292.21	-47306.40
**SRD06**	Skygrid	2013.1811	[2011.5243; 2015.1445]	2022.5963	[2021.8182; 2023.2246]	2021.8495	[2020.7035; 2022.7564]	2014.87	[2013.308; 2016.3548]	-46057.92	-46071.32

eTable 2: Metadata for WNV-L2 sequences obtained from positive samples collected in France between 2022 and 2025. For each sequence, the table provides the region and department of sampling, date of collection, host species, RT-qPCR Ct value (when available), and genome coverage. Host species include mosquitoes (virus obtained from single or pool mosquito homogenates, virus isolated on C6/36 or Vero E6 cells from homogenates, and Molecular Xenomonitoring (MX) samples, *i.e.*, excreta from trapped mosquitoes), birds (*Phoenicopterus* spp., *Serinus canaria*, *Astur gentilis*, *Strix nebulosa, Platycercus eximius, Balearica regulorum, Pica pica, Columba palumbus, Passer domesticus, Streptopelia*

© 2026 Klitting R et al. *JAMA Network Open*.

decaocto), horses (*Equus caballus*), and human cases. Missing values indicate unavailable data. PACA = *Provence-Alpes-Côte d'Azur*; Ct = cycle threshold.

**Sequence ID**	**French Region**	**French department**	**Host species**	**Date of collection**	**Ct**	**Cov**
PV246180	Nouvelle-Aquitaine	Gironde	*Equus caballus*	July 17, 2023	37	>90%
PP482822	Nouvelle-Aquitaine	Gironde	MX	July 25, 2023	17	>90%
PP482824	Nouvelle-Aquitaine	Gironde	MX	July 25, 2023	17	>90%
PP482820	Nouvelle-Aquitaine	Gironde	MX	August 3, 2023	21	>90%
PP482826	Nouvelle-Aquitaine	Gironde	MX	August 24, 2023	19	>90%
PV246187	Nouvelle-Aquitaine	Charente-Maritime	*Phoenicopterus ruber*	July 3, 2023	20	>90%
PV054412	Nouvelle-Aquitaine	Charente-Maritime	*Phoenicopterus chilensis*	July 17, 2023	19	>90%

© 2026 Klitting R et al. *JAMA Network Open*.

PV054413	Nouvelle-Aquitaine	Charente-Maritime	*Phoenicopterus chilensis*	July 17, 2023	24	>90%
PV054410	Nouvelle-Aquitaine	Charente-Maritime	*Phoenicopterus chilensis*	July 27, 2023	25	>90%
PV054411	Nouvelle-Aquitaine	Charente-Maritime	*Phoenicopterus chilensis*	July 27, 2023	25	>90%
PV054414	Nouvelle-Aquitaine	Charente-Maritime	*Phoenicopterus chilensis*	July 31, 2023	21	>90%
PV246186	Nouvelle-Aquitaine	Charente-Maritime	*Platycercus eximius*	August 11, 2023	NA	>90%
PV634346	Nouvelle-Aquitaine	Charente-Maritime	*Platycercus eximius*	August 14, 2023	22	>90%
PV246185	Nouvelle-Aquitaine	Charente-Maritime	*Phoenicopterus ruber*	August 14, 2023	19	>90%
PV634347	Nouvelle-Aquitaine	Charente-Maritime	*Balearica regulorum*	August 20, 2023	24	>90%
PV054415	Nouvelle-Aquitaine	Charente-Maritime	*Astur gentilis*	August 27, 2023	15	>90%

© 2026 Klitting R et al. *JAMA Network Open*.

PV246182	Nouvelle-Aquitaine	Charente-Maritime	*Pica pica*	September 23, 2023	30	>90%
PV246181	Nouvelle-Aquitaine	Charente-Maritime	*Columba palumbus*	September 27, 2023	29	>90%
PV246183	Nouvelle-Aquitaine	Charente-Maritime	*Passer domesticus*	October 3, 2023	17	>90%
PV246184	Nouvelle-Aquitaine	Charente-Maritime	*Streptopelia decaocto*	October 3, 2023	29	>90%
PV054419	Nouvelle-Aquitaine	Charente-Maritime	*Phoenicopterus sp.*	October 4, 2024	24	>90%
PV054416	Nouvelle-Aquitaine	Vienne	*Serinus canaria*	September 12, 2024	21	>90%
PV054417	Nouvelle-Aquitaine	Vienne	*Serinus canaria*	September 12, 2024	18	>90%
OZ263595	Occitanie	Gard	MX	August 16, 2024	27	>90%
OZ261146	Occitanie	Gard	MX	August 29, 2024	15	>90%

© 2026 Klitting R et al. *JAMA Network Open*.

OZ263596	Occitanie	Gard	MX	August 30, 2024	18	>90%
OZ261141	Occitanie	Gard	MX	September 3, 2024	18	>90%
OZ261149	Occitanie	Hérault	MX	August 21, 2024	20	>90%
OZ261142	Occitanie	Hérault	MX	August 28, 2024	19	>90%
OZ261145	Occitanie	Hérault	MX	August 28, 2024	24	>90%
PV054420	Occitanie	Hérault	*Equus caballus*	September 11, 2024	33	>90%
OZ263594	Occitanie	Hérault	MX	September 13, 2024	18	>90%
OZ263593	Occitanie	Hérault	MX	September 17, 2024	32	85%
OZ263592	Occitanie	Hérault	MX	September 30, 2024	32	51%

© 2026 Klitting R et al. *JAMA Network Open*.

PV054409	PACA	Bouches-du-Rhône	*Strix nebulosa*	September 15, 2023	17	>90%
OZ261140	PACA	Var	*Homo sapiens*	September 30, 2022	34	>90%
OZ261148	PACA	Var	MX	August 2, 2024	15	>90%
OZ261144	PACA	Var	MX	August 2, 2024	21	>90%
OZ261143	PACA	Var	*Homo sapiens*	August 23, 2024	26	>90%
OZ261147	PACA	Var	MX	August 23, 2024	25	>90%
OZ313212	Île-de-France	Seine-Saint-Denis	*Homo sapiens*	August 4, 2025	25	>90%
OZ313271	Île-de-France	Seine-Saint-Denis	*Homo sapiens*	July 26, 2025	29	>87%
OZ313260	PACA	Bouches-du-Rhône	*Homo sapiens*	July 18, 2025	30	>80%

© 2026 Klitting R et al. *JAMA Network Open*.

OZ372085	Île-de-France	Seine-Saint-Denis	MX	August 18, 2025	25	>90%
OZ372089	Île-de-France	Seine-Saint-Denis	Single mosquito	August 18, 2025	20.5	>90%
OZ372084	Île-de-France	Val-de-Marne	MX	July 23, 2025	24.5	>90%
OZ372083	Île-de-France	Val-de-Marne	Single mosquito	July 23, 2025	19.6	>90%
OZ372082	Île-de-France	Paris 13e	Single mosquito	September 12, 2025	27.8	>90%
OZ372088	Île-de-France	Paris 13e	Single mosquito	September 12, 2025	25	>90%
OZ372086	Île-de-France	Paris 13e	Single mosquito	September 12, 2025	16.8	>90%
OZ372087	Île-de-France	Paris 13e	Single mosquito	September 12, 2025	29.4	>90%
OZ37209	PACA	Var	*Homo sapiens*			>90%

eMethods

© 2026 Klitting R et al. *JAMA Network Open*.

Mosquito and mosquito excreta collection: MX (Molecular Xenomonitoring) strategy.

Molecular xenomonitoring (MX) was conducted using modified BG-Sentinel or BG-Pro traps (BGS, Biogents AG, Regensburg, Germany), with a protocol adapted from Bigeard et al [1]. In these modified traps, the standard collecting bags were replaced with a 3D-printed MX adapter positioned beneath the intake funnel. The adapter was connected via a conical net and inserted into the depressurized catching pipe. This configuration provided a safe, humid environment for the trapped mosquitoes and included a cotton ball soaked in 10% sugar solution, placed inside an internal feeder to sustain the mosquitoes. An aluminum foil was placed at the bottom of the MX adapter to collect excreta from the mosquitoes during the trapping period. Mosquito collections were conducted for a minimum of 24 hours and up to a maximum of 7 consecutive days. At the end of each trapping period, the trap was reconditioned by removing the MX adapter containing live mosquitoes and replacing it with a fresh one, allowing continuous operation of the trap throughout the surveillance period. Captured mosquitoes were transported to the laboratory and maintained alive inside the MX adapter for at least 24 hours before being frozen at −20 °C for a minimum of 30 minutes. The aluminum foils, containing mosquito excreta, were then carefully removed and sent to partner laboratories for molecular analysis, shipped at room temperature by post. Mosquitoes corresponding to each excreta sample were stored at −20 °C and subsequently processed individually or in pools to detect WNV, only for collections that tested positive for viral RNA in excreta during the initial screening step.

Mosquito excreta and mosquito samples

Mosquitoes and their excreta collected via the MX strategy were tested as previously described [1], with adaptations. Upon arrival at the laboratory, aluminum foils covered with mosquito excreta were stored at 4°C until RNA extraction. For processing, each aluminum foil was carefully rolled and placed at the bottom of a 14 mL plastic tube, then soaked in 800 µL of lysis buffer. The tubes were vortexed vigorously for 2 minutes to ensure thorough mixing. Subsequently, 800 µL of 96–100% ethanol was added, followed by another brief vortexing. The resulting mixture was then transferred onto a silica gel–based RNA extraction column. The type and volume of lysis buffer, as well as the specific extraction kits used, may vary between laboratories. NucleoSpin 96 virus core kit (Macherey-Nagel, Düren, Germany), QIAamp MinElute Virus or QIAamp 96 Virus QIAcube (QIAGEN, Hilden, Germany) kits were used in this study.

Individual mosquitoes were homogenized using 3 mm stainless steel beads in 600 µL of Minimum Essential Medium (MEM) supplemented with 1% penicillin–streptomycin, 1% L-glutamine, 1% kanamycin, and 3% amphotericin B, using a TissueLyser grinder (QIAGEN) for

© 2026 Klitting R et al. *JAMA Network Open*.

2 × 30 seconds at 30 oscillations per second. Viral RNA was extracted with the QIAamp 96 Virus QIAcube HT Kit (QIAGEN) using 100 µL of the individual mosquito lysate.
Nucleic acids from both excreta and mosquitoes were tested by RT-qPCR on a CFX (BIORAD) using WNV primers and probes (ref 001K-05424) and a positive control (ref 001K-05425) from the European Virus Archive Marseille (EVAM). In 2025, RT-qPCR was performed with the Panther Fusion (HOLOGIC) using the same primers and probes. Digital PCR was used to detect WNV from some mosquito excreta samples from the Occitania region.

WNV infection confirmation

Confirmation of WNV infection in humans and horses, individuals were considered positive if they met at least one of the following criteria: (i) direct detection of viral RNA by PCR in blood or organ samples; (ii) serological evidence of recent infection, defined as seroconversion from IgM^+/IgG^- to IgM^+/IgG^+ or a fourfold increase in IgG titer between two consecutive samples; or (iii) positive IgM serology in cerebrospinal fluid (CSF) or (iv) successful virus isolation.

Animal samples

Organs from horses (brain) and organs (brain, liver and spleen) and swabs (oral and/or cloacal) from birds were tested by RT-qPCR at the French National Reference Laboratory for West Nile virus (ANSES, Maisons-Alfort). Briefly, 100 mg of each organ sample was homogenized in 1 mL of Dulbecco's Modified Eagle Medium (DMEM) using the FastPrep system (MP Biomedicals). Swab heads were cut and incubated in 300 µL of DMEM for 10 minutes, with intermittent vortexing for 30 seconds. After incubation, the swab heads were removed while retaining as much of the eluate as possible.
Organ homogenates and swab eluates were centrifuged at 2,000 × g for 5 minutes at 4°C. RNA was extracted from 100 µL of the resulting supernatant using the ID Gene™ Mag Universal Extraction Kit, following the manufacturer's instructions. Duplex RT-qPCR assays targeting both WNV and the β-actin gene were performed using a QS5 thermocycler and the AgPath-ID™ One-Step RT-PCR kit (Thermo Fisher Scientific), with 5 µL of extracted RNA per reaction. Primers and probes were supplied by the European Union Reference Laboratory for Equine Diseases (EURL Equine Diseases).

Viral isolation

Viral isolation was attempted on African green monkey kidney cells (Vero E6, ATCC C1008) and *Aedes albopictus* insect cells (C6/36, ATCC CRL-1660). Individual mosquito homogenates were filtered using 0·5 mL PVDF ST ultra-free-cl millipore (Merck, Darmstadt, Germany) and diluted (1/8) in 350 µL of MEM (for Vero E6 cells) or Leibovitz's L15 medium (for C6/36 cells)

© 2026 Klitting R et al. *JAMA Network Open*.

supplemented with 2.5% fetal bovine serum (FBS), 1% penicillin–streptomycin, 1% L-glutamine, 1% Kanamycin, and 3% Amphotericin B, before inoculation on confluent culture of Vero E6 and C6/36 on 6-well flat bottom cell culture plates. Individual mosquito homogenate inocula were incubated 1 hour at 37°C in a 5% CO2 atmosphere (Vero E6 cells) or 28°C without CO2 (C6/36 cells) to infect cells mono-layers prior to be removed and replaced by 4 mL of MEM (for Vero E6 cells) or L-15 (for C6/36 cells) supplemented with 7% heat-inactivated FBS, 1% penicillin–streptomycin, 1% L-glutamine, 1% Kanamycin, and 3% Amphotericin B. Cell cultures were examined daily for the presence of cytopathic effect (CPE).A volume of 200 µL supernatant of post-infection day 5 was tested using a specific WNV RT-qPCR assay as described above (WNV primers and probes (ref 001K-05424) and a positive control (ref 001K-05425) provided by EVAM).

Virus sequencing

Virus sequencing at LNR at the French National Reference Laboratory for West Nile virus

RNA extracted from WNV-positive samples was selected for whole genome sequencing. Two sequencing approaches were employed: (i) For a subset of samples, RNA was directly used for RNA-seq library preparation using the Illumina Stranded Total RNA Prep kit, with indexing performed using IDT for Illumina indexes. All reagents were sourced from Illumina (91000 Évry, France). Libraries were sequenced on a NextSeq 2000 system (Illumina) using a P2 flow cell (2 × 300 cycles). (ii) Other RNA samples were processed using an amplicon-based sequencing approach as described by Quick *et al.* [2], with primers specifically designed to generate 400-bp overlapping amplicons covering the full genome of WNV lineage 2. These primers were provided by the European Union Reference Laboratory (EURL) for Equine Diseases.

Briefly, 10 µL of RNA was reverse-transcribed using the SuperScript™ IV VILO™ Master Mix (Invitrogen, Thermo Fisher Scientific, USA), which includes random hexamers, according to the manufacturer's instructions. Then, 2 µL of the resulting cDNA was amplified using the lineage-specific primer pools and the Q5® High-Fidelity 2X Master Mix (New England Biolabs, MA, USA), following both manufacturer instructions and the protocol by Quick *et al.* [2]. PCR products were pooled and purified with the HighPrep™ PCR Clean-up System (MagBio Genomics Inc., USA), using a 1.8× bead-to-sample ratio and eluted in 35 µL of nuclease-free water. DNA concentration was measured using the Qubit™ dsDNA High Sensitivity (HS) or Broad Range (BR) assay kits (Invitrogen, Thermo Fisher Scientific, USA).

Amplicons were then prepared for sequencing on either Illumina or Oxford Nanopore Technologies (ONT) platforms. For Illumina sequencing, libraries were prepared using the DNA Prep kit and corresponding indexes, and sequenced on a NextSeq 2000 using a P2

reagent kit (2 × 300 cycles). For Nanopore sequencing, libraries were prepared following the "Ligation Sequencing Amplicons – Native Barcoding Kit 24 V14" protocol (version NBA_9168_v114_revR_30Jan2025), using the SQK-NBD114.24 kit (Oxford Nanopore Technologies, UK). A 1.5× bead ratio was maintained throughout the library preparation steps. 100 femtomoles of the library were loaded onto a FLO-MIN114 (R10.4.1) flow cell, and sequencing was performed for 72 hours using standard settings in MinKNOW software (version 24.11.10).

Raw read sequences obtained with Illumina sequencing were cleaned using fastp 0.23.2 [23], low quality reads (Q<20) were filtered out, then assembled into contigs with the Spades 3.15.5 assembler [3]. Cleaned reads were aligned with genes using bwa-mem2 version 2.2.1. Consensi were obtained using samtools 1.14, seqtk 1.3 (https://github.com/lh3/seqtk), and bcftools 1.15 [4].

Nanopore raw data were first basecalled and demultiplexed using MinKNOW embedded DORADO software (version 7.6.18) and the Super Accurate model. Raw reads were then analyzed using a custom mapping workflow on Geneious Prime (version 2022.0.2). Briefly, primer labels were removed from the read sequences (29 bases removed in 5' and 3' of each read), size selection was applied to select reads with the expected amplicon length (between 150 and 500 bp). Trimmed and filtered reads were then mapped on reference sequences using minimap2 (kmer length of 10). Consensus sequences were produced with a minimum of 30 of sequencing depth.

Virus sequencing at the National Reference Center for Arboviruses

WNV genomes from human, and mosquito (excreta, whole mosquito) samples were sequenced at the NRC using an amplicon-based sequencing approach with a set of 8 overlapping amplicons, previously used for WNV sequencing [1]. These primers generate eight overlapping amplicons across the full viral genome via two multiplexed PCR reactions. Reverse transcription and amplification were performed using the SuperScript™ IV One-Step RT-PCR System with Platinum™ SuperFi DNA Polymerase (Thermo Fisher Scientific, USA). PCR reactions (25 µL final volume) consisted of 3 µL of nucleic acid extract, 1.25 µL of each primer (10 µM), 12.5 µL of 2× RT-PCR Master Mix, 0.5 µL of SuperScript IV RT mix, and 6.5 µL of RNase-free water. Thermocycling conditions were as follows: reverse transcription at 55 °C for 10 min, initial denaturation at 98 °C for 2 min, followed by 40 cycles of 98 °C for 10 s, 55 °C for 10 s, and 68 °C for 1 min 45 s, with a final extension at 68 °C for 5 min.

The size of PCR amplicons was verified by agarose gel electrophoresis. For each sample, an equimolar pool of all PCR products was generated and subsequently purified using the

© 2026 Klitting R et al. *JAMA Network Open*.

Monarch® PCR & DNA Cleanup Kit (New England Biolabs, USA). DNA quantification was performed using the Qubit® dsDNA HS Assay Kit with the Qubit 2.0 fluorometer (Thermo Fisher Scientific, USA). Purified amplicons were fragmented to an average size of ~250 bp using a Bioruptor® sonicator (Diagenode, Liège, Belgium). Fragmented DNA was then used for library construction with the Ion Plus Fragment Library Kit on the AB Library Builder™ System (Thermo Fisher Scientific), following the manufacturer's protocol.

To ensure accurate equimolar pooling of barcoded libraries, quantitative real-time PCR was performed using the Ion Library TaqMan™ Quantitation Kit (Thermo Fisher Scientific). Equimolar pools of libraries were subjected to emulsion PCR, and the resulting products were loaded onto Ion 530™ chips using the Ion Chef™ Instrument (Thermo Fisher Scientific). Sequencing was conducted on the Ion S5™ System (Thermo Fisher Scientific) according to the manufacturer's instructions.

After demultiplexing, read data were analyzed with an in-house Snakemake pipeline. Reads were first trimmed using cutadapt (v4.4) to remove amplification primers and with trimmomatic (v0.39) [5] to remove short and low quality reads. Read alignment was achieved using BWA MEM (v0.7.17) using, as a reference, the best match identified by blasting (magicblast, v1.7.7 [28]) sequencing reads using a database of flavivirus sequences including reference sequences representative of the genetic diversity of WNV. Consensus sequences were called using the ivar (v1.3.1) consensus command [6] with a minimum coverage depth of 50x.

All genomic sequences were made publicly available on GenBank and/or Pathoplexus.

Phylogenetic analysis

Publicly available sequences representative of the genetic diversity of WNV-L2 were retrieved from the pathoplexus database (SeqSet doi:10.62599/PP_SS_67.2). Sequences were trimmed to their coding regions (ORFs), aligned using MAFFT (v7.511), and manually inspected in AliView (v1.0). Phylogenetic relationships between public WNV genomes and sequences generated in this study were inferred using a maximum likelihood (ML) approach implemented in IQ-TREE (v1.6.12). The best-fit nucleotide substitution model was selected using ModelFinder, and branch support was evaluated with ultrafast bootstrap approximation (UFBoot2), using 1,000 replicates. Based on the initial phylogenetic tree, we selected a subset of 218 sequences representing the Cluster A of WNV-L2. Sequences from France and the sequence from Spain (GB accession: OZ221437) were excluded from the subsampling process. We first performed model selection based on a previous version of the dataset that did not include the 2025 sequences (2 sequences from the Paris area and one from the eastern Mediterranean area). We used two different substitution models (the HKY substitution model

© 2026 Klitting R et al. *JAMA Network Open*.

with a gamma-distributed rate variation among sites and no partition into codon positions (HKYG4), or the Shapiro-Rambaut-Drummond-2006 (SRD06)), an uncorrelated lognormal (UCLN) clock model, and three different coalescent models (constant, exponential and bayesian skygrid). We ran single MCMC chains of 100 million states and performed marginal likelihood estimation using path sampling/ stepping-stone sampling. We used Tracer (v1.753) for inspecting the convergence and mixing, discarding the first 10 % of steps as burn-in, and ensuring that estimated sampling size (ESS) values associated with estimated parameters were all >200. Based on the best-fit model (the SRD06 substitution model, with an UCLN clock model, and a bayesian skygrid coalescent model, see supplementary table 1), using the final dataset, we ran three MCMC chains of 100 million states, using Tracer for inspecting convergence as described above. All alignment, xml, and tree files for this study are available on a private repository [7]. Final phylogenetic trees were visualized using the ggtree R package.

eReferences

1. Bigeard C, Pezzi L, Klitting R, et al. Molecular Xenomonitoring (MX) allows real-time surveillance of West Nile and Usutu virus in mosquito populations. *PLoS Negl Trop Dis*. 2024;18(12):e0012754. doi:10.1371/journal.pntd.0012754

2. Quick J, Grubaugh ND, Pullan ST, et al. Multiplex PCR method for MinION and Illumina sequencing of Zika and other virus genomes directly from clinical samples. *Nat Protoc*. 2017;12(6):1261-1276. doi:10.1038/nprot.2017.066

3. Bankevich A, Nurk S, Antipov D, et al. SPAdes: a new genome assembly algorithm and its applications to single-cell sequencing. *J Comput Biol*. 2012;19(5):455-477. doi:10.1089/cmb.2012.0021

4. Danecek P, Bonfield JK, Liddle J, et al. Twelve years of SAMtools and BCFtools. *Gigascience*. 2021;10(2):giab008. doi:10.1093/gigascience/giab008

5. Bolger AM, Lohse M, Usadel B. Trimmomatic: a flexible trimmer for Illumina sequence data. *Bioinformatics*. 2014;30(15):2114-2120. doi:10.1093/bioinformatics/btu170

6. Grubaugh ND, Gangavarapu K, Quick J, et al. An amplicon-based sequencing framework for accurately measuring intrahost virus diversity using PrimalSeq and iVar. *Genome Biol*. 2019;20(1):8. doi:10.1186/s13059-018-1618-7

7. Klitting R. Dataset rklitting github. https://github.com/rklitting/WNV_USUV_NouvelleAquitaine_2023

© 2026 Klitting R et al. *JAMA Network Open*.
