# Supplementary material for: Genomic Epidemiology of West Nile Virus in Paris
Source: JAMA Netw Open. 2026 Feb 16;9(2):e2559588. doi: 10.1001/jamanetworkopen.2025.59588 (PMC12910402; doi:10.1001/jamanetworkopen.2025.59588)
Supplement: Supplement 2. — Data Sharing Statement [file jamanetwopen-e2559588-s002.pdf]

Data Sharing Statement

Klitting. Genomic Epidemiology of West Nile Virus in Paris. *JAMA Netw Open*. Published February 16, 2026. doi:10.1001/jamanetworkopen.2025.59588

Data
Data available: Yes
Data types: Other (please specify)
Additional Information: All virus sequences are accessible either on GenBank (accessions specified in the text) or on Github (https://github.com/rklitting/WNV_phylo_2024-2025). The xml and tree files used in this study are available on Github (same repository as above). The R code used to create the graphical representation is available as supplementary material.
How to access data: Github (https://github.com/rklitting/WNV_phylo_2024-2025). Pathoplexus and GenBank
When available: With publication

Supporting Documents
Document types: Statistical/analytic code
How to access documents: https://github.com/rklitting/WNV_phylo_2024-2025
When available: With publication

Additional Information
Who can access the data: Raphaelle Klitting; Albin Fontaine
Types of analyses: genomic epidemiology
Mechanisms of data availability: without investigator support
Any additional restrictions: NA
